# Use of the Strengths and Difficulties Questionnaire in child and school health services among children aged 4 and 6 years in Southern Norway: clinical considerations

**DOI:** 10.1186/s12887-023-03837-1

**Published:** 2023-01-19

**Authors:** Eirin Mølland, Kristin Haraldstad, Eirik Abildsnes, Åshild Tellefsen Håland, Unni Mette Stamnes Köpp, Liv Fegran, Thomas Westergren

**Affiliations:** 1 Department of Economics and Finance, School of Business and Law, University of Agder, PO Box 422, 4604 Kristiansand, Norway; 2 NORCE, Kristiansand, Norway; 3 Department of Health and Nursing Science, Faculty of Health and Sport Sciences, University of Agder, Kristiansand, Norway; 4 Kristiansand Municipality, Kristiansand, Norway; 5 Department of Psychosocial Health, Faculty of Health and Sport Science, University of Agder, Kristiansand, Norway; 6 Department of Children and Adolescents Mental Health (ABUP), Sørlandet Hospital HF, Kristiansand, Norway; 7 Department of Peadiatrics, Sørlandet Hospital HF, Kristiansand, Norway

**Keywords:** Child health, Mental health, Primary healthcare, School health services, Maternal-child health centers, Cutoff, Patient reported outcome measures

## Abstract

**Background:**

Parent reported mental health can be assessed by the Strengths and Difficulties Questionnaire (SDQ). Currently, Norwegian norms for parent-reported SDQ do not exist, whereas Swedish, Danish, and United Kingdom (UK) norms have been published. We aimed to (1) describe parent-reported SDQ among children aged 4 and 6 years in Southern Norway, (2) evaluate empirical cutoff values within the context of the Starting Right^TM^ project in relation to the Swedish, Danish, and UK cutoffs, and (3) evaluate the representativeness of the study sample with regard to parental socioeconomic status.

**Methods:**

This study included parent-reported observations for 665 children (63% consent rate). Means and standard deviations were calculated for the domains of SDQ, and gender differences were assessed. Based on the Swedish, Danish, and UK cutoffs and the 80^th^ and 90^th^ percentile cutoff values within the study, we calculated the total number of children with borderline and abnormal scores.

**Results:**

Boys had higher mean total difficulties (7.3 vs 5.6) and impact scores (0.3 vs 0.1) and lower prosocial scores (8.3 vs 8.8) than girls. The differences in means were largest in the case of externalizing symptoms (5.0 vs 3.6) and hyperactivity subscore (3.2 vs 2.3). Using the UK cutoff values, 28 and 25 children had borderline and abnormal total difficulties scores, respectively. The corresponding numbers using the within study or Scandinavian cutoff values were 84–99 and 54–79, respectively. Overall, our study sample was well representative of the target population.

**Conclusions:**

Our findings consistently indicated that girls had better SDQ scores than boys among children aged 4 and 6 years. Fewer children would be identified as having mental health difficulties using the UK cutoff values than using the Scandinavian age- and gender-relevant cutoff values.

## Background

In Norway, child health clinics are well developed, free of charge, organized within the municipal primary health care system, and expected to safeguard and promote the healthy development of children. However, a recent review of national practices in the Nordic countries and Scotland revealed that none of these countries fulfilled the World Health Organization’s screening criteria for developmental surveillance ensuring feasibility and quality of the instruments used [1]. Universal screening may improve the early identification of problems, create opportunities for systematic evaluation, facilitate user involvement and interdisciplinary collaboration, and improve service quality [2–5]. Growth and weight monitoring are implemented nationally in child and school health services in Norway [6]. However, parent- or child-reported measures and screening of development, mental health, and quality of life are rarely recorded in the child health service systems [1]. The importance of screening is related to the fact that unhealthy trajectories, including mental health problems and problem behaviors as well as poor physical health, may start early during childhood [7–9]. Problems are often related to adverse circumstances, such as physical and emotional abuse, neglect, and household dysfunction [10], and challenging family trajectories [10]. Investments in early interventions for improved child health have greater returns both for the society and children at risk [11] and should rely on the evidence-based practice logic of assessing the problem before intervening [12]. Therefore, valid and reliable instruments for early identification and evaluation need to be integrated into routine child health services [7, 12]. Moreover, the instruments need to be feasible, acceptable, and affordable for the users (children, parents, and professionals), and facilitate understanding, dialogue, and engagement concerning challenges without labelling the child or family [7].

In Sweden [13] and Scotland [14], such efforts concerning the mental health of children have been initiated using instruments such as the Strengths and Difficulties Questionnaire (SDQ). The SDQ assesses mental health symptoms using 25 items representing internalizing symptoms (peer and emotional problems), externalizing symptoms (conduct problems and hyperactivity), and prosocial behavior. In addition, an impact score that reflects how these symptoms influence daily life is assessed using five items [15]. The SDQ can be reported by both parents and teachers of children aged ≥2 years as well as by children aged ≥11 years [15].

The Norwegian Starting Right^TM^ project, which is an innovation that provides public health nurses with an online tool and practical routines for the assessment of children’s health and development using parent- and child-reported questionnaires, has been piloted in child and school health services in Agder County in Southern Norway [16]. To assess children’s mental health, the parent-reported SDQ is used for children aged ≥4 years. The current study hence includes population-based data on children reported through ordinary child health services, needed to further develop and improve routine use of SDQ in child health clinics.

Country-specific normative data are important in psychosocial research because psychosocial functioning is country- and culture-specific [17]. Currently, Norwegian norms for the SDQ do not exist [18], whereas Swedish [19], Danish [20], and United Kingdom (UK) [21] norms have been published or provided on the SDQ information website [20, 21]. The SDQ cutoff values rely on the 80^th^ percentile for the presence of borderline mental health problems and the 90^th^ percentile for abnormal mental health problems [21]. Large population-based studies have been conducted in Norway in different regions and age ranges, and they demonstrated appropriate psychometric properties [18]. Because of the lack of Norwegian norms and cutoff values, the UK norms were used in the Starting Right™ project. However, Swedish [19] and Danish [20], as well as Norwegian [18] studies, suggested lower cutoff values than the UK norms using the 80^th^ and 90^th^ percentiles. Distributions of scores were highly similar across the Nordic countries [22]. Lower cutoffs may increase the sensitivity and identify more children with challenges. Based on sensitivity/specificity analysis, Sveen et al. [23] recommended a total difficulty cutoff score ≥10 when using the SDQ in the general population. However, using a large population-based cohort, the developers of the SDQ reported that no threshold for psychopathology was found [24]. Hence, based on the reported correlation between the SDQ and psychopathology across the full range of scores, they suggested employing the SDQ as a dimensional measure [24].

Differently from previous studies, data used in this study is collected through routine follow-up in the Norwegian child health clinics. To develop clinical use of the parent-reported SDQ in preventive child health care by public health nurses, we therefore aimed to (1) describe parent-reported SDQ among children aged 4 and 6 years in Southern Norway, (2) evaluate empirical cutoff values within the context of the Starting Right™ project in relation to the Swedish, Danish, and UK cutoffs, and (3) evaluate the representativeness of the study sample with regard to parental socioeconomic status.

## Methods

### Study design

The Starting Right™ project provides an observational longitudinal population-based cohort from Agder County in Southern Norway based on ordinary consultations in the child and school health services. However, the present study recruited children aged 4 and 6 years from three urban and rural municipalities.

### Data collection

Data were collected using an online tool (CheckWare, CheckWare Ltd., Trondheim, Norway) between May 2019 and May 2021. Nine days before the scheduled consultation between the child and school health nurse, a text message with a secure link was sent to the parents of the child, whose phone number was registered in the child’s journal. Parents then logged in through the secured Norwegian public e-services login system (ID-porten). First, parents of all children were asked to respond to the SDQ parent questionnaire. Then, only the parents of 6-year-old children were asked to respond to the KIDSCREEN-27 questionnaire, which assesses the parent-measured health-related quality of life (data not included in the present study). All parents were asked to respond to the questionnaires for clinical use to inform the public health nurse about their child’s health. A report was generated for the public health nurse for each instrument concerning each child individually. After responding to the questionnaires concerning their child’s health, parents were provided with written information regarding the research project and the possibility for consent, including alignment of the data with Norwegian statistics concerning parental education, income, and ethnic background.

### Participants

Our data consisted of 732 parent-reported SDQ questionnaires for children aged 4 and 6 years (consent rate, 63%). In a few cases (*n* = 13), multiple responses were provided for the child by the same parent. In these cases, we used the first response. In addition, there were 50 cases where both parents reported the SDQ for their child. In these cases, we included the report from “parent 1,” because the majority of our SDQ reports were from “parent 1”; in most cases, this was the mother. Thus, a total of 665 individual parent-reported SDQs were finally used.

### Socioeconomic background

The data were linked with administrative data from Statistics Norway. We were able to identify nearly all fathers’ and mothers’ data in the administrative data, only those of nine fathers and five mothers were not identified. *Parental education* was measured as the highest level of education completed by 2020 for mothers and fathers individually. *Parental income* was measured in 2019. Our data included the following two different measures of income: income from employment and household income after tax per consumption unit calculated using the European Union equivalence scale. Our data did not include the absolute income values but included the parents’ relative ranks in the income distribution relative to the population of Norway. Statistics Norway data include percentiles for different income measures for all individuals aged > 16 years living in Norway; these data are separately provided for men and women. Our data provide information about which percentile the parents belonged to. Finally, our data also included information about the *children’s immigration status*.

### The Strength and Difficulties Questionnaire

The SDQ parent questionnaire comprises 20 items representing a total difficulties score, additional five items representing prosocial behavior, and an impact supplement of eight items of which five constitutes the impact score according to the scoring manual [21]. Difficulties and prosocial items are described as e.g., “Generally liked by other children” with a response format from “Not true” to “Somewhat true,” and “Certainly true.” The total difficulties score compounds an internalizing symptoms score consisting of peer and emotional problems with five items each and an externalizing symptoms score consisting of hyperactivity and conduct problems with five items each [15]. Each item is scored as 0, 1, or 2 and each domain will thereby range from 0 to 10, internalizing and externalizing symptoms scores from 0 to 20, and the total difficulties score from 0 to 40. The impact score is based on five of the items with a response format ranging from “Not at all” to “Only a little” (both 0), “Quite a lot” (1), and “A great deal” (2). Hence, it also ranges from 0 to 10. The UK norms and cutoff values, referring to the 80^th^ and 90^th^ percentiles for borderline and abnormal values, respectively, are provided separately for age groups 2–4 years and 4–17 years, but not separately for different genders [20]. The Swedish [19] and Danish [20] values applied in the present study are distinct between the genders and cover the age groups 4–5 years and 5–7 years, respectively. In general, the suggested cutoff values from the UK are higher than those from the Scandinavian countries, leading to fewer identified cases if applied.

### Statistical analysis

We performed descriptive analysis using STATA (StataCorp. 2019, Stata Statistical Software: Release 16. College Station, TX, USA). Means and standard deviations (SDs) were computed. The differences in means between boys and girls were computed and analyzed using independent samples *t* tests assuming unequal variance (Welch’s test). The 95% confidence intervals (CIs) for the mean difference were calculated. Based on the suggested cutoff values from Sweden, Denmark, and the UK, we calculated the total number of respondents in the borderline and abnormal groups in our study. Finally, based on the present sample, we calculated the 80^th^ and 90^th^ percentiles for cutoff values within our study population.

## Results

### Descriptive statistics

In total, parents reported individual observations for 320 girls (48%) and 345 boys (52%); 89% of responses were from mothers. All observations were provided for children either 4 years old (70%) or 6 years old (30%). Among the children, 47% and 51% were girls at 4 and 6 years, respectively. The mean age of the mothers and fathers was 35.2 years (SD, 4.6) and 37.6 years (SD, 5.7), respectively.

### SDQ scores reported by parents

The mean total difficulties score reported by parents was 6.5 (SD, 4.2) whereas internalizing and externalizing symptoms scores were 2.2 (SD, 2.2) and 4.3 (SD, 3.1), respectively (Table 1). The total prosocial score was 8.5 (SD, 1.5) and the impact score was 0.2 (SD, 0.8). Parents reported significantly fewer total difficulties symptoms in girls than boys (mean difference, ˗1.7 [95% CI { ˗2.3, ˗1.1}]). The major difference between girls and boys was found in the case of externalizing symptoms (˗1.4 [95% CI { ˗1.9, ˗0.9}]), whereas no significant difference was observed in internalizing symptoms. Externalizing symptoms of hyperactivity (˗0.9 [95% CI { ˗1.2, ˗0.6}]) and conduct problems (˗0.5 [95% CI { ˗0.7, ˗0.3}]) were both lower in girls than boys. Peer symptoms were also marginally lower in girls than boys (˗0.2 [95% CI { ˗0.4, ˗0.1}]). Moreover, parents reported higher prosocial score (0.5 [95% CI {0.2, 0.7}]) and lower impact score (˗0.2 [95% CI { ˗0.4, ˗0.1}]) in girls than boys. The emotional symptoms score was the only difficulties score (in addition to the collapsed internalizing symptoms score) that was similar between girls and boys in this study population.

**Table 1:** Mean SDQ values (distinct for girls and boys)

	(1)	(2)	(3)	(4)
	Full Sample	Girls	Boys	Difference
Total difficulties	6.457	5.572	7.278	˗1. 706^***^
	(4.248)	(3.616)	(4.616)	[˗2.334, ˗1.079]
Internalizing	2.167	2.006	2.316	˗0.310
	(2.158)	(1.933)	(2.341)	[˗0.635, 0.0157]
Emotional	1.460	1.422	1.496	˗0.0738
	(1.525)	(1.460)	(1.585)	[˗0.305, 0.158]
Peer symptoms	0.707	0.584	0.820	˗0.236^*^
	(1.204)	(1.062)	(1.313)	[˗0.417, ˗0.0550]
Externalizing	4.290	3.566	4.962	˗1.397^***^
	(3.101)	(2.667)	(3.320)	[˗1.853, ˗0.940]
Conduct	1.558	1.303	1.794	˗0.491^***^
	(1.437)	(1.226)	(1.574)	[˗0.705, ˗0.277]
Hyperactivity	2.732	2.263	3.168	˗0.906^***^
	(2.204)	(1.962)	(2.327)	[˗1.232, ˗0.579]
Prosocial	8.537	8.778	8.313	0.465^***^
	(1.519)	(1.312)	(1.658)	[0.239, 0.692]
Impact	0.211	0.084	0.328	˗0.243^***^
	(0.781)	(0.450)	(0.979)	[˗0.358, ˗0.129]
*N*	665	320	345	665

Columns 1, 2, and 3: Mean values, standard deviation in parentheses. Column 4: Mean differences, 95% confidence interval using t-test assuming unequal variance ^*^ *p* < 0.05, ^**^ *p* < 0.01, ^***^ *p* < 0.001

#### Cutoff values

Table 2 presents suggested cutoff values that have been developed based on population samples from Sweden, Denmark, and the UK and the 80^th^ and 90^th^ percentiles in the present Norwegian study (10^th^ and 20^th^ percentiles for the prosocial score). The consequences of differences in the cutoff values are illustrated in Table 3, where we have calculated the number of individuals in our sample that were categorized as having borderline and abnormal scores using the different suggested cutoff values. The numbers of individuals with both borderline and abnormal scores were substantially lower on all SDQ dimensions using the cutoff values from the UK than those obtained using the cutoff values from Sweden/Denmark or the 80^th^ and 90^th^ percentiles in the present study. Considering the total difficulties score, using the UK cutoff values, approximately 4% of our sample was categorized as having borderline scores and approximately 4% as having abnormal scores. However, using the Swedish cutoff values, the proportion of our sample that was categorized as having borderline and abnormal scores was 13% and 12%, respectively. Using the Danish cutoff values, 15% and 8% of our sample were categorized as having borderline and abnormal scores, respectively. Because the 80^th^ and 90^th^ percentile cutoff values in the present study were rounded to the nearest integer, the proposed cutoff values from the present study would result in the categorization of 84 (13%) and 74 (11%) children as having borderline and abnormal scores, respectively. Using the cutoff proposed by Sveen et al. [23], 134 (20%, 44 girls and 90 boys) children in our study would be categorized as having a score indicating emotional and/or behavioral problems.

**Table 2:** Country specific cutoff values for borderline and abnormal parent-reported SDQ-scores

	UK 2–4/4–17 years [20]		Sweden 4/5 years [18]		Denmark 5–7 years [19]		Norway **4 years/6 years**^**1**^	
	Borderline	Abnormal	Borderline	Abnormal	Borderline	Abnormal	Borderline	Abnormal
Total difficulties	13/14	16/17					**10/9**	**12/12**
Boys			9/8	10/11	10	14	**10/11**	**14/14**
Girls			10/9	13/12	9	12	**9/7**	**11/9**
Emotional symptoms	3/4	4/5					**2/3**	**3/4**
Boys			2/2	3/3	4	5	**2/3**	**4/4**
Girls			2/2	3/3	4	5	**2/3**	**3/4**
Peer problems	3/3	4/4					**1/1**	**2/2**
Boys			2/2	3/2	2	3	**2/2**	**2/2**
Girls			1/1	2/2	2	3	**1/1**	**2/2**
Conduct problems	4/3	5/4					**3/2**	**4/3**
Boys			4/3	4/4	3	4	**3/3**	**4/4**
Girls			3/3	4/4	3	4	**3/2**	**3/2**
Hyperactivity	6/6	7/7					**5/5**	**6/6**
Boys			4/4	5/5	5	7	**5/5**	**6/6**
Girls			3/3	5/4	4	6	**4/3**	**5/4**
Prosocial score^2^	6/5	5/4					**7/7**	**6/7**
Boys			6/7	5/6	6	5	**7/7**	**6/6**
Girls			7/8	6/6	7	6	**8/8**	**7/7**

^1^Based on the 80^th^ and 90^th^ (10^th^ and 20^th^ for the prosocial score) percentiles from the present study from Agder County, Norway. ^**2**^For prosocial score, cutoff values are based on the 10^th^ and 20^th^ percentiles

**Table 3:** Total number and proportion of children categorized as having borderline and abnormal scores

	UK [20]		Sweden [18]		Denmark [19]		Norway ^1^	
	Borderline	Abnormal	Borderline	Abnormal	Borderline	Abnormal	Borderline	Abnormal
	Sum/ratio	Sum/ratio	Sum/ratio	Sum/ratio	Sum/ratio	Sum/ratio	Sum/ratio	Sum/ratio
Total difficulties	28	25	85	79	99	54	84	74
	0.042	0.038	0.128	0.119	0.149	0.081	0.126	0.111
Emotional symptoms	49	57	138	120	30	38	129	84
	0.074	0.086	0.208	0.180	0.045	0.057	0.194	0.126
Peer problems	29	24	82	98	77	53	50	130
	0.044	0.036	0.123	0.147	0.116	0.080	0.075	0.195
Conduct problems	48	31	51	66	87	66	46	128
	0.072	0.047	0.077	0.099	0.131	0.099	0.069	0.192
Hyperactivity	31	41	114	154	110	58	83	102
	0.047	0.062	0.171	0.232	0.165	0.087	0.125	0.153
Prosocial	32	26	87	46	61	39	102	100
	0.048	0.039	0.131	0.069	0.092	0.059	0.153	0.150
*N*	665	665	665	665	665	665	665	665

The table shows the total number and proportion of children categorized as borderline and abnormal when suggested cutoff values from the UK, Sweden and Denmark were applied, respectively. The corresponding cutoff values are presented in Table 2. We used the age-appropriate cutoff values and separated between genders if the cutoff values were different. For the UK cutoff values, the norms for children aged 2–4 years were applied to the 4-year-old children in our study. For the Swedish cutoff values, the norms for 4- and 5-year-old children were applied. For the Danish cutoff values, the norms for children aged 5–7 years were applied. In cases where the cutoff values were similar for the borderline and abnormal scores, we only included individuals in the abnormal group. ^1^The Norwegian numbers are based on the 80^th^ and 90^th^ (10^th^ and 20^th^ for the prosocial score) percentiles from the present study

#### Representativity

**Fig. 1:**
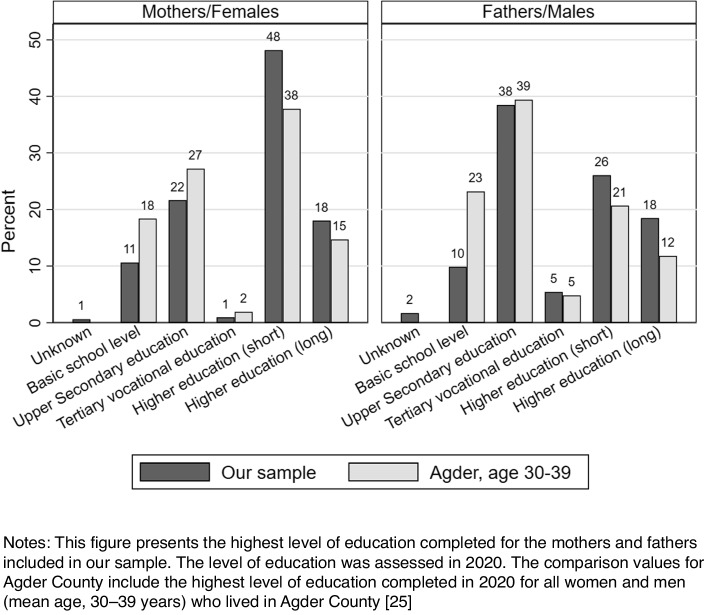
Comparison of education level for parents in the present study and individuals in Agder County

A comparison of the highest level of education completed for parents in our study against the official statistics for individuals living in Agder County is presented in Fig. 1. The comparison age group of 30–39 years was selected because it included the mean age for the parents included in our study. In general, both mothers and fathers included in our study had all levels of education, i.e., the included children had parents with both low (basic education level completed) and higher education. However, the proportion of parents who had only completed basic school level (9 or 10 years) was lower in our sample than the average proportion of such individuals in Agder County. Moreover, our sample included a higher proportion of parents with higher education (bachelor’s degree as well as master’s degree or higher) than the average proportion of such individuals in Agder County.

**Fig. 2:**
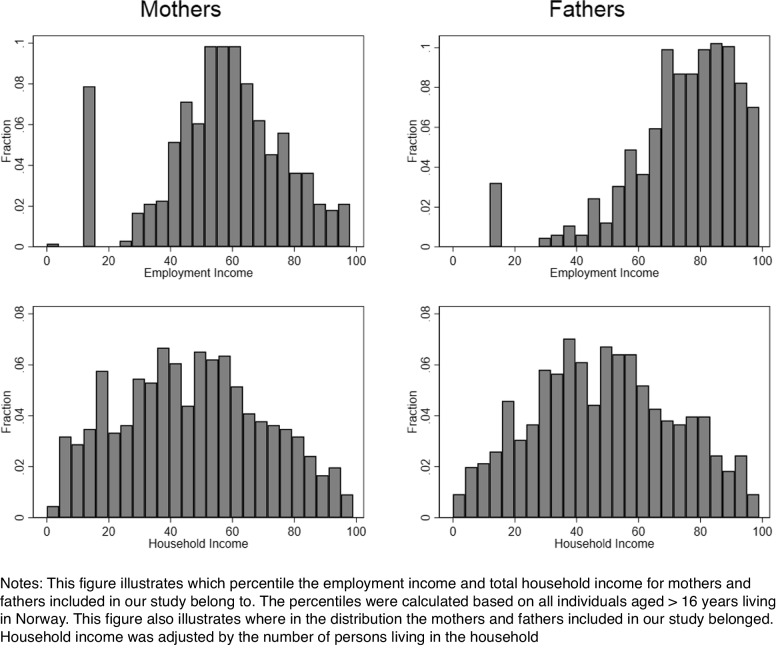
Employment and household income levels for mothers and fathers, 2019

Figure 2 presents the relative income level for the parents in our sample. Among fathers, the majority belonged to the 60^th^ percentile or higher and the employment income distribution was skewed to the right. Among the mothers included in our study, few individuals belonged to the highest percentiles. If we had a 100% representative population, the bars in the income distribution would have been equally high. However, this was not expected because we only included parents and not all adults aged ≥16 years, and the mean age for the mothers and fathers was 35.2 and 37.6 years, respectively. Household income, which was corrected for the number of persons in the household, was more evenly distributed, including observations of parents from the lowest percentiles to the highest.

**Fig. 3:**
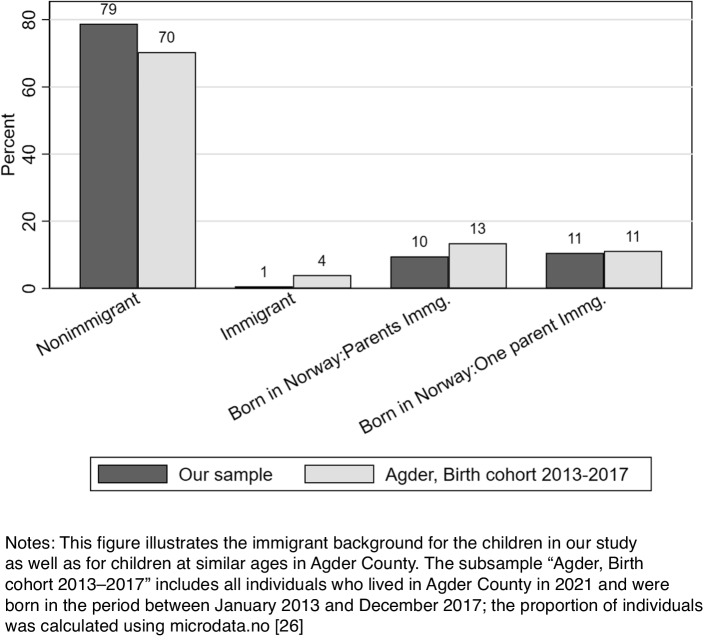
Immigrant background of the children in this study compared to reference population

In our study, we managed to include children with and without immigrant backgrounds (Fig. 3). Compared with the Agder County population, our sample consisted of more individuals from the nonimmigrant population (79% vs 70%). The proportion of children in our study who were born in Norway with one or two parents being immigrants was similar to this proportion for children in the same age group in Agder County. In our study, 1% of children had immigrated to Norway whereas that proportion was 4% in the corresponding age group in Agder County population in 2021.

## Discussion

The aims of the present study were to develop the clinical use of the parent-reported SDQ in preventive child health care by public health nurses, describe the parent-reported SDQ, evaluate empirical cutoff values within the context of the Starting Right™ project in relation to the Swedish, Danish, and UK cutoffs, and evaluate the representativeness of the study sample with regard to parental socioeconomic status.

Our main findings showed that boys had higher total difficulties and impact scores than girls. The differences in means between boys and girls were largest in the case of externalizing symptoms and hyperactivity subscore. However, girls had higher prosocial scores than boys. Our findings consistently indicated that girls had better SDQ scores than boys in the included age group. Moreover, fewer children would be identified as having mental health challenges using the UK cutoff values than using the Scandinavian cutoff values. Applying the 80^th^ percentile cutoffs in the present study, 158 of 665 children were identified as having mental health difficulties.

The mean parent-reported total difficulties scores in the present study were nearly similar to those reported in previous Norwegian [18] and Nordic studies [22]. Even though Norwegian norms and cutoffs are not established [18], the availability of country-specific normative data is of interest in psychosocial research because psychosocial functioning is known to be country- and culture-specific [17]. In the Starting Right™ project, the UK cutoffs were used to guide the child and school health nurses’ interpretation of individual results. As shown in the results, the nurses may overlook more children (approximately 67%) with or at risk of mental health problems using the UK cutoffs than using the Swedish/Danish cutoffs or the “in-study” 80^th^ percentile cutoffs (Table 3).

Even though cutoffs can be difficult to establish, the SDQ total difficulties score correlates with mental health challenges at the full range of scores [24]. Moreover, the use of different concepts related to what SDQ measures are reported in the literature; Sveen et al. [23] used emotional and behavioral disorders whereas Goodman [15] used the concept of “a total difficulties score.” Consequently, an interpretation by a clinician may be either in the direction of using the score and a cutoff to diagnose the child (or hypothesize a diagnosis and refer to specialist health care), or to gain insight into a child’s mental health symptoms as perceived by the parent of the child. Moreover, screening may be the first step in a dialogue and provide valuable insight into children’s mental health. Use of the SDQ may facilitate communication between family/child and the public health nurse, and areas of concern can be identified and discussed. Such knowledge will be important to make decisions regarding the child’s needs and possible support strategies. For clinical use, gender and population-based 80^th^ and 90^th^ percentile cutoffs may help the clinician to focus their efforts on understanding and supporting the children with the most mental health symptoms, which represents a different approach than categorizing the children in terms of psychopathology.

Although Kornør and Heyerdahl [18] did not make the abovementioned distinction clear, they suggested that the parent-reported SDQ should not be used to screen for psychopathology, which would refer to a clinical use of the SDQ for diagnosing disorders. Nevertheless, they emphasized the importance of a low cutoff if the SDQ is to be used in municipal services with a low incidence of mental disorders, which may apply for the current study. However, a recent systematic review concluded that the SDQ demonstrated predictive validity for language and behavioral concerns in preschool-aged children in a community setting [27].

Sveen et al. [23] suggested a Norwegian cutoff score ≥10 to determine psychopathology at age 4 years with satisfactory sensitivity and specificity. However, in their follow-up study of children aged 6 years, they reported many nonpersistent cases and a rather low positive predictive value (9.5%) and a high negative predictive value (99.6%) [28]. Such findings would practically mean that children at 4 years of age with low or unproblematic scores are at low risk of mental health problems 2 years later, while children identified with problems at 4 years of age may not have persistent problems 2 years later. Hence, the clinical implications may be in line with the recommendations made by Kornør and Heyerdahl [18], to not use the parent-reported SDQ as a tool for suggesting or predicting psychiatric disorders. In line with this finding, a Danish study among preschool children reported that the SDQ was useful for screening at a preschool age to identify children at an increased risk of mental health problems. However, the authors emphasized that early screening with the SDQ predictive algorithms cannot stand alone, and repeated assessments of children are needed, especially regarding internalizing mental health problems [29]. Screening tools rely on predictive validity and may imply the risk of false positive and/or false negative cases [1]. Applying the 90^th^ percentile from the current study as a cutoff (≥ 12, see Table 2) for abnormal range in the study population of Sveen et al. [28] would have led to a sensitivity of 54%, which would implicate many cases missed. Our study has a limited contribution for establishing Norwegian norms and cutoffs for diagnostic purposes, whereas the novelty concerns the study of clinical anamnestic considerations. In the current study context, screening is not used to decide who is receiving follow-up from the public health nurses, but to inform the content of the follow-up and strengthen the dialog between the public health nurses and the family. For instance, public health nurses may guide parents about how to relate to children with different difficulties reported by the parents using the SDQ instrument. Our study’s 80^th^ percentile, as well as the study by Sveen et al. [28], indicate that public health nurses should pay attention to all children with a total difficulties score above 9 or 10. Nevertheless, context and age specific cutoffs to guide content of follow-up in primary health care need further investigation.

Moreover, Nilsen et al. [10] reported that internalizing and externalizing mental health problems can be traced to as early as 18 months of age. However, they did not measure mental health problems using the SDQ. In addition, children who show early signs of mental health problems and have mothers who receive the appropriate support can change their trajectory in a healthier direction [10]. A plausible clinical implication would then be to increase or facilitate support to parents of children with the most symptoms. The latter may support a clinical use of the SDQ, not to diagnose or predict diagnosis and problems, but to adjust the effort of the primary child health care providers to individuals at current risk.

In our study, parents overall reported more total difficulties symptoms among boys than girls, particularly driven by externalizing symptoms and the subdomain of hyperactivity. This finding is in line with those of earlier studies. In a Dutch study, boys (aged 4–5 years) scored higher than girls on the hyperactivity and total difficulties domains, and more boys than girls scored in the clinical range of prosocial behavior [17]. Hence, public health nurses should be aware that gender differences could represent the different needs of children.

The mental health of a child may follow certain trajectories but also vary through age. An important factor determining mental health according to the Norwegian TOPP study is personality, and how the environment of the child challenges, or reacts/responds to, the child’s needs [10]. A recent policy statement of the American Academy of Pediatrics also highlighted the importance of personalizing the response to individual children’s needs when facing any adversity [30]. Hence, the SDQ may represent an outcome mostly relevant to the child (and family) from a subjective perspective. Because children are born with different personalities and temperaments, and may face adversity in different environments, they also need different nurturing responses from their environments for their healthy development [7, 10, 31].

Asking children and families about the children’s symptoms, acknowledging and being responsive to the child’s personality and needs, facilitating reduction of family stress, and helping the parents to incorporate core skills, as suggested by Garner and Yogman [30], may represent a feasible and suitable use of the SDQ in primary health care. In such a context, cutoffs may be used as advisory instead of definers and as markers of unhealthy trajectories and/or reflections of adversity. Advisory cutoffs may then motivate efforts in child primary health care on children’s own premises.

### Representativeness of the study sample

Our study sample represented all groups of socioeconomic status and immigrant backgrounds; however, it had a minor overrepresentation of parents with higher education and Norwegian background. Nevertheless, 11% of mothers and 10% of fathers had basic school level education (9–10 years of schooling) only.

Representativity in population-based studies may often be a challenge. Public health nurses have also raised concerns about whether all types of families can be included in the Starting Right™ project and if responses will be received from the immigrant population owing to barriers due to language skills or technical issues (smartphone and the secure ID). Our findings indicated that few responses (1%) were received from parents of children in cases where both parents and the child were born outside Norway and had since immigrated to Norway. However, in general, the proportion of children in the population who had immigrated was rather low (4%). For children born in Norway, we had a relatively representative sample including children with and without immigrant backgrounds.

### Strength and limitations

The consent rate to this study was 63%, which we consider relatively high. However, it is a limitation that we did not have information about the group not consenting to this research. Data from the included individuals indicated that they represented children with different socioeconomic backgrounds both in terms of education and income, which is a strength of this study. However, a limitation is that our income data included the percentile of income compared with the income of the entire population aged > 16 years, while our study population only included mothers and fathers with a mean age of 35.2 years and 37.6 years, respectively. Responses from mothers were overrepresented because in most cases, the health centers only had the mothers’ phone numbers. Hence, the text message may only have been sent to the mother. Furthermore, despite that the current study corresponds with previous Scandinavian studies concerning identification rates, we cannot tell if the children in need of follow-up are identified. However, the instrument is implemented through ordinary services and all children receive individual follow-up by the public health Nurses.

### Implications

Advisory cutoffs of the SDQ, relying on the Scandinavian, but not on the UK, norms may be used to reflect children’s individual and present needs in Norway, and help public health nurses to personalize their care and focus on children and families with the highest needs. Parent-reported SDQ in children aged 4 and 6 years can be representatively collected in municipal health services using an online tool. Furthermore, Norwegian SDQ norms and cutoffs should be further developed.

## Conclusions

Our findings indicate that girls had better SDQ scores than boys among children aged 4 and 6 years in Southern Norway, as measured using parent-reported SDQ. Approximately only 1out of 3 children would be identified as having mental health difficulties using the UK cutoff values (*n* = 53) compared to using the Scandinavian age- and gender-relevant cutoff values (*n* = 153–164). Overall, the study sample was well representative of the population in the region.

## Data Availability

The datasets generated and analysed during the current study are not publicly available due to regulation by the Norwegian Data Protection Authority but are available from the corresponding author on reasonable request.

## Abbreviations

CI: Confidence interval
SD: Standard deviation
SDQ: Strengths and Difficulties Questionnaire
UK: United Kingdom
